# Apoptosis-related genes-based prognostic signature for osteosarcoma

**DOI:** 10.18632/aging.204042

**Published:** 2022-05-03

**Authors:** Fei Yang, Yi Zhang

**Affiliations:** 1 Department of Orthopaedics, Zibo Central Hospital, Zibo 255036, Shandong, China

**Keywords:** osteosarcoma, risk score, apoptosis-related genes, prognosis

## Abstract

Osteosarcoma (OS) is a common malignant primary tumor of skeleton, especially in children and adolescents, characterized by high lung metastasis rate. Apoptosis has been studied in various tumors, while the prognostic role of apoptosis-related genes in OS has been seldom studied. Three OS related datasets were downloaded from Gene Expression Omnibus (GEO) database. Univariate Cox and LASSO Cox regression analysis identified optimal genes, which were used for building prognostic Risk score. Subsequent multivariate Cox regression analysis and Kaplan-Meier survival analysis determined the independent prognostic factors for OS. The immune cell infiltration was analyzed in CIBERSORT. Basing on 680 apoptosis-related genes, the OS patients could be divided into 2 clusters with significantly different overall survival. Among which, 6 optimal genes were identified to construct Risk score. In both training set (GSE21257) and validation set (meta-GEO dataset), high risk OS patients had significantly worse overall survival compared with the low risk patients. Besides, high Risk score was an independent poor prognostic factor for OS with various ages or genders. Three immune cells were differentially infiltrated between high and low risk OS patients. In conclusion, a six-gene (TERT, TRAP1, DNM1L, BAG5, PLEKHF1 and PPP3CB) based prognostic Risk score signature is probably conducive to distinguish different prognosis of OS patients.

## INTRODUCTION

Osteosarcoma (OS) is a common malignant primary tumor of skeleton, especially in children and adolescents, which is usually characterized by developing sarcoma cells to immature bone or osteoid tissue in metaphysis regions of long bones [1–3]. Along with the development of clinical practices, the overall survival rate of non-metastatic OS patients treated with specialized surgery and chemotherapy has been improved to 50%-70% [4]. Whereas, it has been estimated that there is already micro-metastasis at the diagnosis in more than 50% OS patients [5]. Meanwhile, high aggressiveness and lung metastasis rate of OS are still great challenges limiting survival rate of OS patients [6], leading to a poorer survival rate of approximately 20-30% [7–9]. Immunosuppressive feature of OS is also important in causing undesirable prognosis of patients [10]. Additionally, those OS patients at a similar clinical stage with same treatments might have different outcomes, due to the individual genetic and tumor heterogeneity [11, 12]. Accordingly, deepening mechanisms underlying the progression and prognosis of OS should be further explored, besides, more reliable and clinically meaningful diagnostic or prognostic signatures are urgently demanded for OS.

Increasing genes or signatures have been reported as biomarkers for OS, contributing to improve the outcome of OS patients. For example, targeting BMPR2 and HIF1-α has been indicated to promisingly prevent metastasis and progression in OS patients [7]. A recent study has revealed an immune-related genes based prognostic signature for OS patients, which could also predict the survival of OS patients [13]. Moreover, another risk signature-based on three metastasis-associated genes has been suggested to predict the prognosis of OS patients [14]. However, to the best of our knowledge, the prognostic role of apoptosis-related genes in OS has been seldom studied. Apoptosis, as a way of programmed cell death, is catalyzed by numerous proteins’ proteolytic cleavage, especially affected by the enzymatic activity of effector caspases 3, 6 and 7, etc. [15, 16]. The apoptotic process comprises in multiple steps, including nuclear membrane breakdown, membrane blebbing, transformation genomic DNA into nucleosomal structures, and so on [17, 18]. Currently, some anti-OS drugs have been indicated to involve in the apoptosis. Apatinib has been evidenced to facilitate the apoptosis in OS patients via regulating VEGFR2/STAT3/BCL-2 signaling, and thereby inhibiting the growth of OS [19]. Moreover, Metformin might have antitumor potential in OS patients, through inducing apoptosis and autophagy via influencing ROS/JNK signaling [20]. Estrogen receptor β (ERβ) is widely involved in the apoptosis and autophagy, which might contribute to suppress the proliferation and metastasis of OS cells [21]. The overexpression of GRIM-19 has been proved to promote the radiation-induced apoptosis of OS cells [22]. Collectively, it will be of great importance to explore the prognostic role of apoptosis-related genes in OS, which would be conducive to those high risk OS patients’ personalized management.

In our study, we herein aimed to explore the prognostic role of apoptosis-related genes in OS patients, basing on the public OS data in Gene Expression Omnibus (GEO) database and the corresponding comprehensive bioinformatics analysis, attempting to construct a reliable prognostic signature for OS. Our findings are expected to give more conducive information for indirect improvement of the prognosis of OS patients.

## MATERIALS AND METHODS

### Data sources

We totally downloaded 3 datasets from the GEO database (Gene Expression Omnibus) (https://www.ncbi.nlm.nih.gov/geo/). In GSE21257, mRNA chip data of 53 OS patients was obtained, whose complete clinical survival information was shown in Table 1. Moreover, in GSE16091 and GSE39058 datasets, there were 34 and 41 OS samples and corresponding clinical information, respectively. The data in GSE16091 and GSE39058 datasets were analyzed on Affymetrix Human Genome U133A Array and Illumina HumanHT-12 WG-DASL V4.0 R2 expression beadchip platforms, respectively. Besides, GSE16091 and GSE39058 were merged as meta-GEO dataset.

**Table 1:** Clinicopathological characteristics of osteosarcoma patients from GSE21257 database.

**Characteristics**		**Patients(N=53)**	
		**NO.**	**%**
**Gender**	Female	19	35.85%
	Male	34	64.15%
**Age**	≤16(Median)	25	47.17%
	>16(Median)	28	52.83%
**Grade**	I	13	24.53%
	II	16	30.19%
	III	13	24.53%
	IV	5	9.43%
	Unknown	6	11.32%
**Survival Time**	Long(>5 years)	6	11.32%
	Short(<5 years)	47	88.68%
**OS status**	Dead	23	43.40%
	Alive	30	56.60%

### Cluster analysis

Those OS patients with complete survival information were clustered according to “K-mean” method, using R language (version 4.1.0, the same below).

### LASSO Cox regression analysis

Next, the OS samples were subjected to the univariate Cox regression analysis based on the expression data to screen the prognosis related genes (significance threshold P <0.01). The LASSO Cox regression analysis was then conducted to optimize the prognosis related genes, using glmnet of R [23]. The optimal genes and the following formula were used for the Risk score calculation of all OS samples. In below formula, Coefi referred to the risk coefficient of each factor in the LASSO-Cox model, and Xi represented the gene expression value in our study. The best cutoff value of Risk score was determined by using survival, survminer and two-sided log-rank test of R. All OS samples were divided in high and low risk groups according to the cutoff value.


$$Risk\;Score=\sum_{i=1}^{n}Coef_{i\;}*X_{i}$$

### Survival analysis

The overall survival rate was estimated basing on Kaplan-Meier method in survival and survminer package of R. The significance of difference among various groups was tested by log-rank test. Multivariate Cox regression analysis was used to find all independent prognostic factors for OS patients.

### Nomogram building

Nomogram is usually used to predict the prognosis of cancer. All independent prognostic factors were contained to build Nomogram to predict the 1-year, 2-year and 3-year overall survival of OS patients, utilizing rms package of R. The calibration curves were used to assess the prognostic predictive performance of Nomogram.

### The relative proportion of immune cell infiltration

The relative infiltrated proportions of various immune cells in each OS sample were estimated using software CIBERSORT [24]. In CICERSORT, basing on gene expression matrix and preset 547 barcode genes, the immune infiltrating cell composition was characterized according to deconvolution algorithm. The estimated infiltrated proportions of every sample sum up to 1.

### The expression of crucial immune checkpoints

The correlation between some key immune checkpoints’ expression (CTLA4, PDL1, LAG3, TDO2) and the Risk score in OS samples was studied. Besides, immune checkpoints’ expression was also compared between high and low risk OS patients.

### Availability of data

All data generated and analyzed in this study are available from the Gene Expression Omnibus (GEO, https://www.ncbi.nlm.nih.gov/geo/) database.

## RESULTS

### OS patients with different prognosis could be divided basing on apoptosis related genes

**Figure 1:**
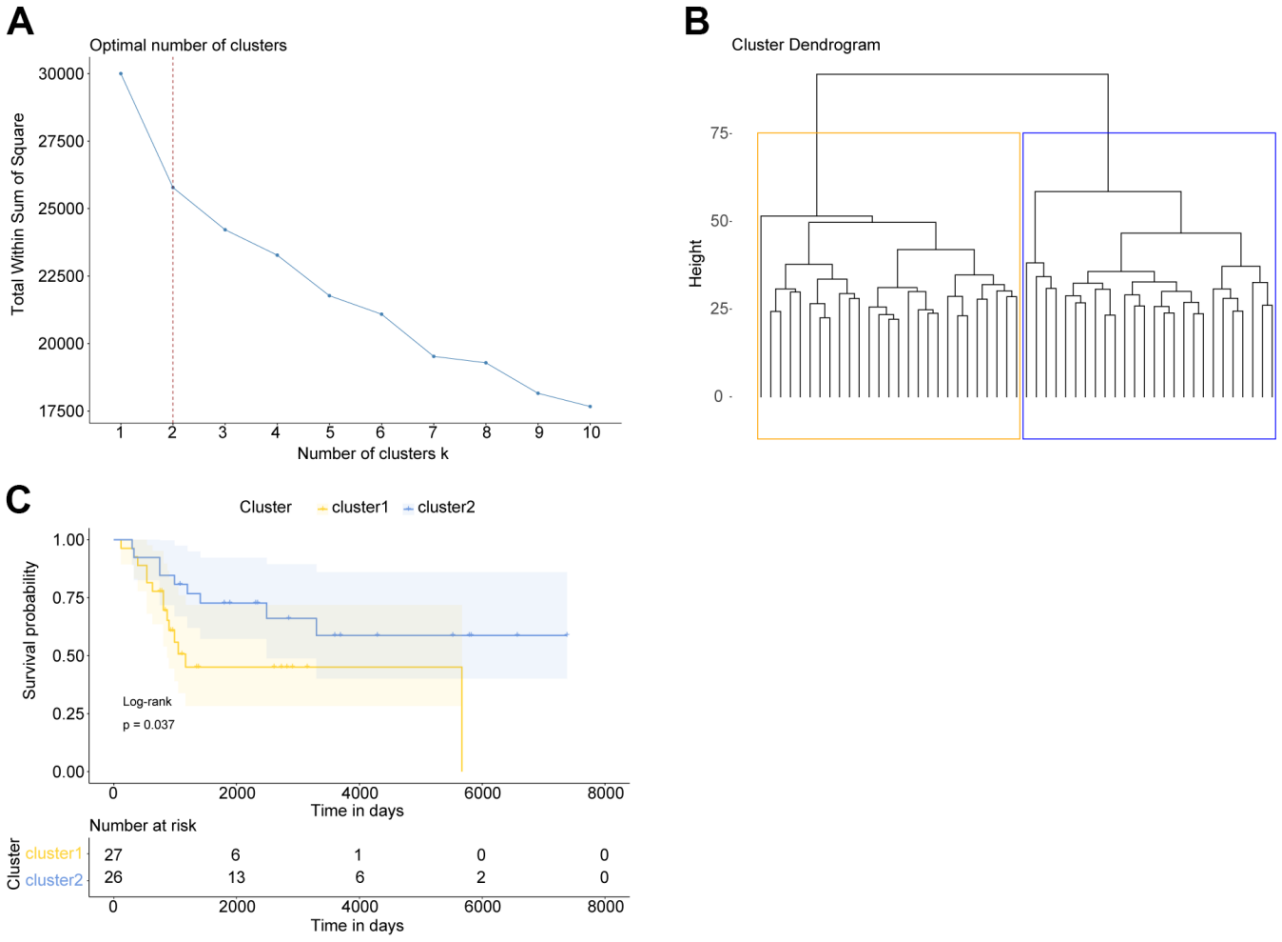
**OS patients with different prognosis could be divided basing on apoptosis-related genes.** (**A**) Elbow diagram indicated that the optimal number of clusters was k=2. (**B**) The cluster results of OS samples. (**C**) Kaplan Meier survival curve of OS patients in two clusters. P was calculated according to log-rank test.

Firstly, we have downloaded totally 680 apoptosis-related genes from GO (Gene Ontology) and KEGG (Kyoto Encyclopedia of Genes and Genomes) datasets in GSEA database (Supplementary Table 1). Then, basing on these 680 apoptosis-related genes, the OS samples in GSE21257 dataset were clustered according to “K-mean” method using R. Sum of the squared errors (SSE) results indicated that the optimal number of clusters was k=2 (Figure 1A), and all OS samples were clustered into 2 categories (Figure 1B). Kaplan-Meier survival analysis suggested that the OS patients in these two clusters had significantly different overall survival (Figure 1C).

### Prognostic Risk score model for OS patients

**Figure 2:**
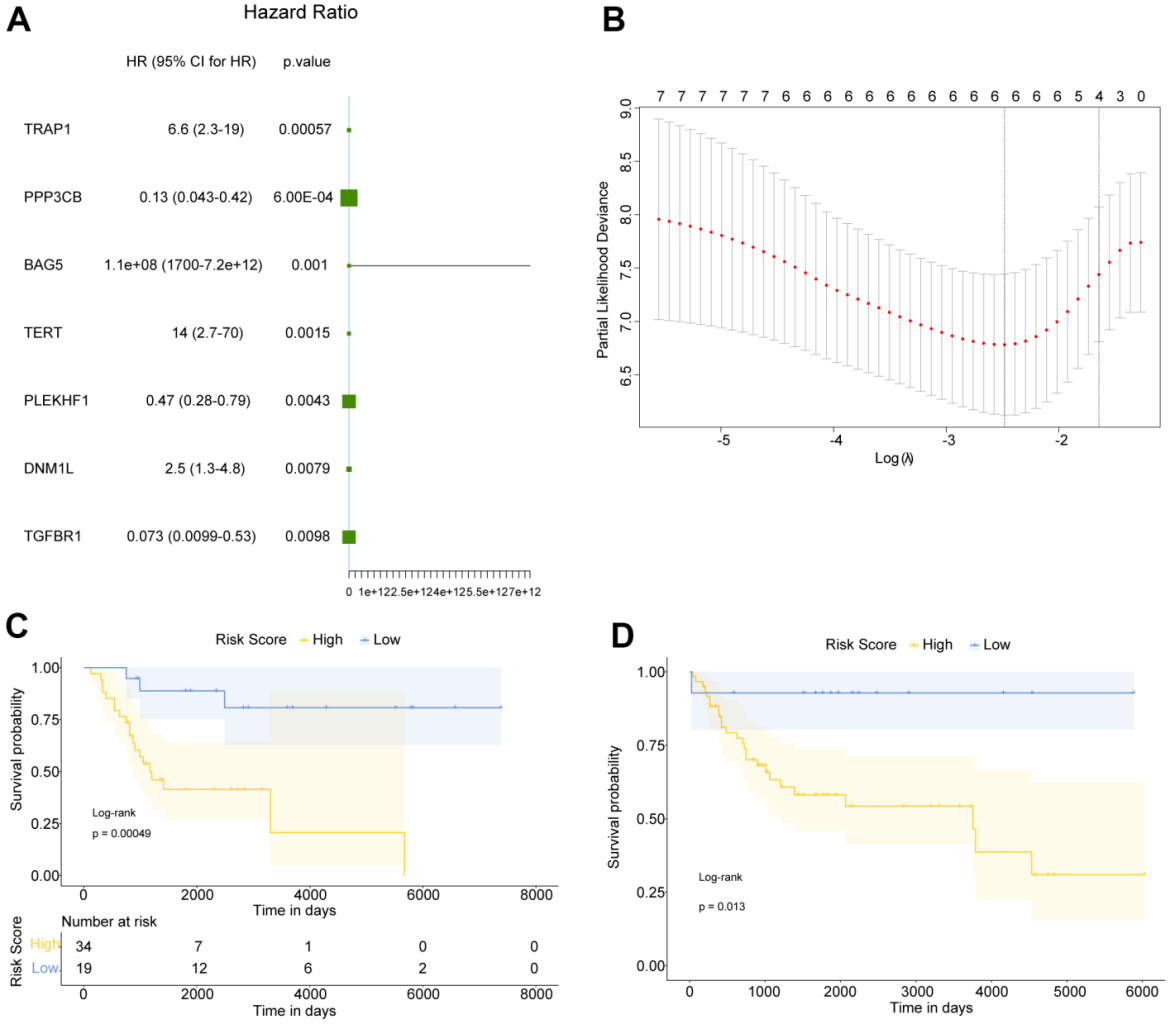
**Construction of prognostic Risk score for OS patients.** (**A**) Totally 7 genes were significantly related to the prognosis of OS. HR: Hazard ratio; 95% CI: 95% confidence interval. (**B**) The smallest lambda in LASSO Cox regression analysis was corresponding to the optimal number of genes. (**C**, **D**) Kaplan Meier survival curves of OS patients in GSE21257 and meta-GEO datasets, respectively. P was calculated based on log-rank test.

Taking 680 apoptosis-related genes’ expression as continuous variables, the OS samples in GSE21257 dataset were then subjected to an univariate Cox regression analysis. All apoptosis-related genes’ Hazard ratio (HR) were calculated, among which 7 genes were screened with P value <0.01 (Figure 2A). The subsequent LASSO Cox regression analysis was conducted on these 7 genes, during which lowest lambda value was corresponding to the optimal number of genes (Figure 2B). The 6 optimal genes comprised TERT, TRAP1, DNM1L, BAG5, PLEKHF1 and PPP3CB.

Subsequently, in GSE21257 and meta-GEO (containing GSE16091 and GSE39058) datasets, the mean value and standard deviation (SD) of these 6 genes’ expression value were normalized to 0 and 1, respectively. Then the normalized expression was weighted with the regression coefficient in LASSO Cox regression analysis to establish a prognostic Risk score for OS patients, Risk Score = (0.19843870*TERT)+(0.61052614*TRAP1)+ (0.09921504*DNM1L)+ (0.19947814*BAG5)+ (-0.30350396*PLEKHF1)+ (-0.33883222*PPP3CB). Accordingly, the Risk score of each OS patient was calculated, and the OS samples in GSE21257 and meta-GEO were divided into high and low Risk score groups basing on best cutoff value (-0.627). The survival analysis was then performed on high and low risk OS patients in GSE21257 and meta-GEO, the results of which suggested that in both two datasets, high risk OS patients had poorer overall survival compared with low risk patients (Figure 2C, 2D). Therefore, our data indicated that our Risk score, based on TERT, TRAP1, DNM1L, BAG5, PLEKHF1 and PPP3CB, had a relatively good prognostic predictive performance.

### Independent prognostic factor for OS patients -- Risk score

**Figure 3:**
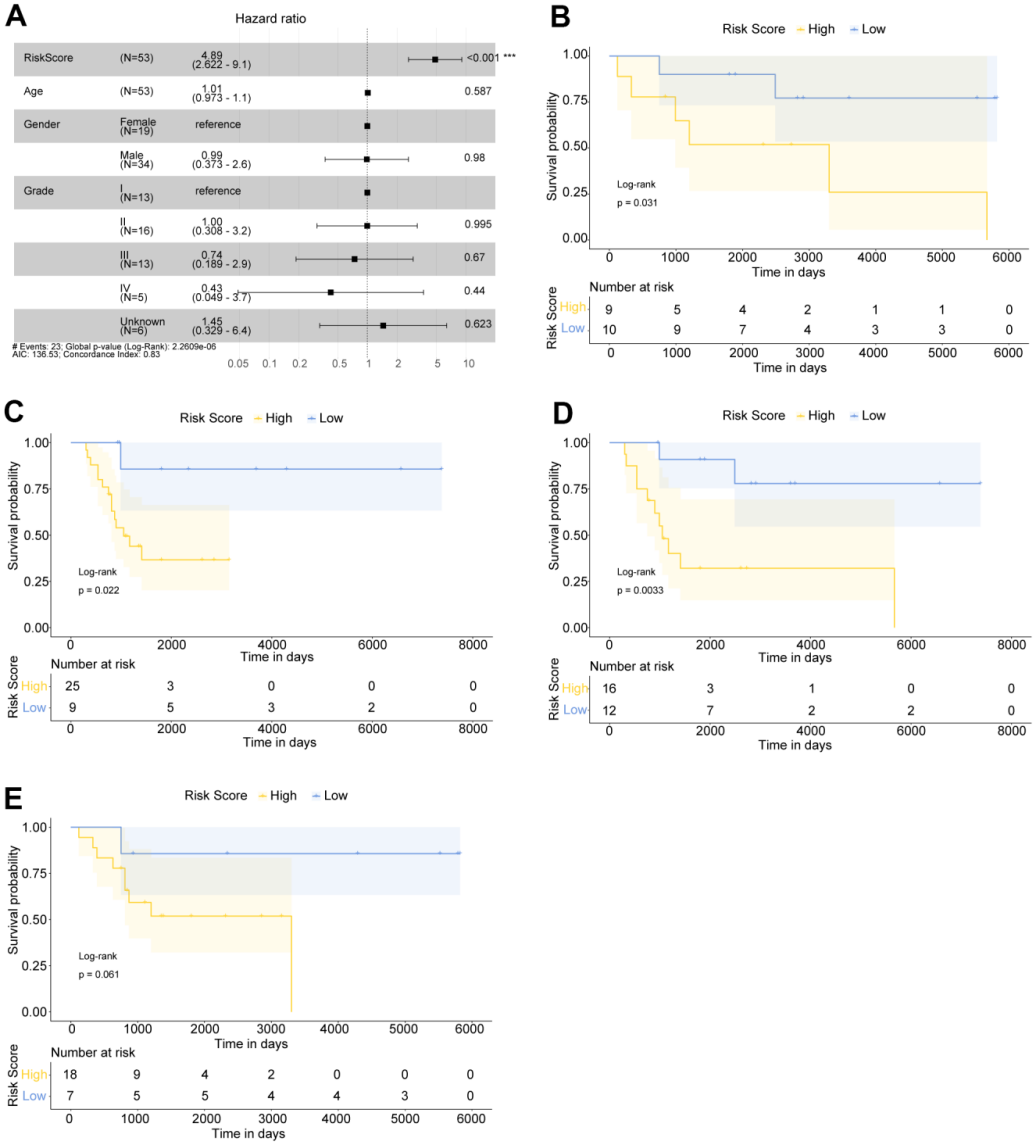
**The Risk score was an independent prognostic indicator for OS patients.** (**A**) Multivariate Cox regression analysis indicated that Risk score was significantly related to the overall survival of OS patients. OS patients with Hazard ratio (HR) >1 had higher death risk, and those with HR <1 had lower risk. (**B**, **C**) Kaplan Meier survival curves of female and male OS patients, respectively. (**D**, **E**) Kaplan Meier survival curves of >16 year-old and ≤16 year-old OS patients, respectively.

A multivariate Cox regression analysis was performed, containing age, gender, stage and Risk Score, in order to explore the independent prognostic factors for OS patients (Figure 3A). We found that the Risk score was still significantly correlated with overall survival of patients, besides high Risk score was a poor prognostic indicator for OS patients (HR=4.89, 95% CI: 2.622-9.1, P <0.001).

To further explore the prognostic value of Risk score in OS patients with different ages or genders, we have also conducted Kaplan-Meier survival analysis on different types of patients. The results suggested that in female (Figure 3B), male (Figure 3C), and >16 years old OS patients (Figure 3D), all high risk OS samples had significantly worse overall survival compared with low risk patients. In ≤16 years old patients, high risk OS samples generally tended to have poorer overall survival compared with low risk patients (Figure 3E). Our findings implied that Risk score was a promising independent prognostic factor for OS patients.

### Nomogram for OS patients

**Figure 4:**
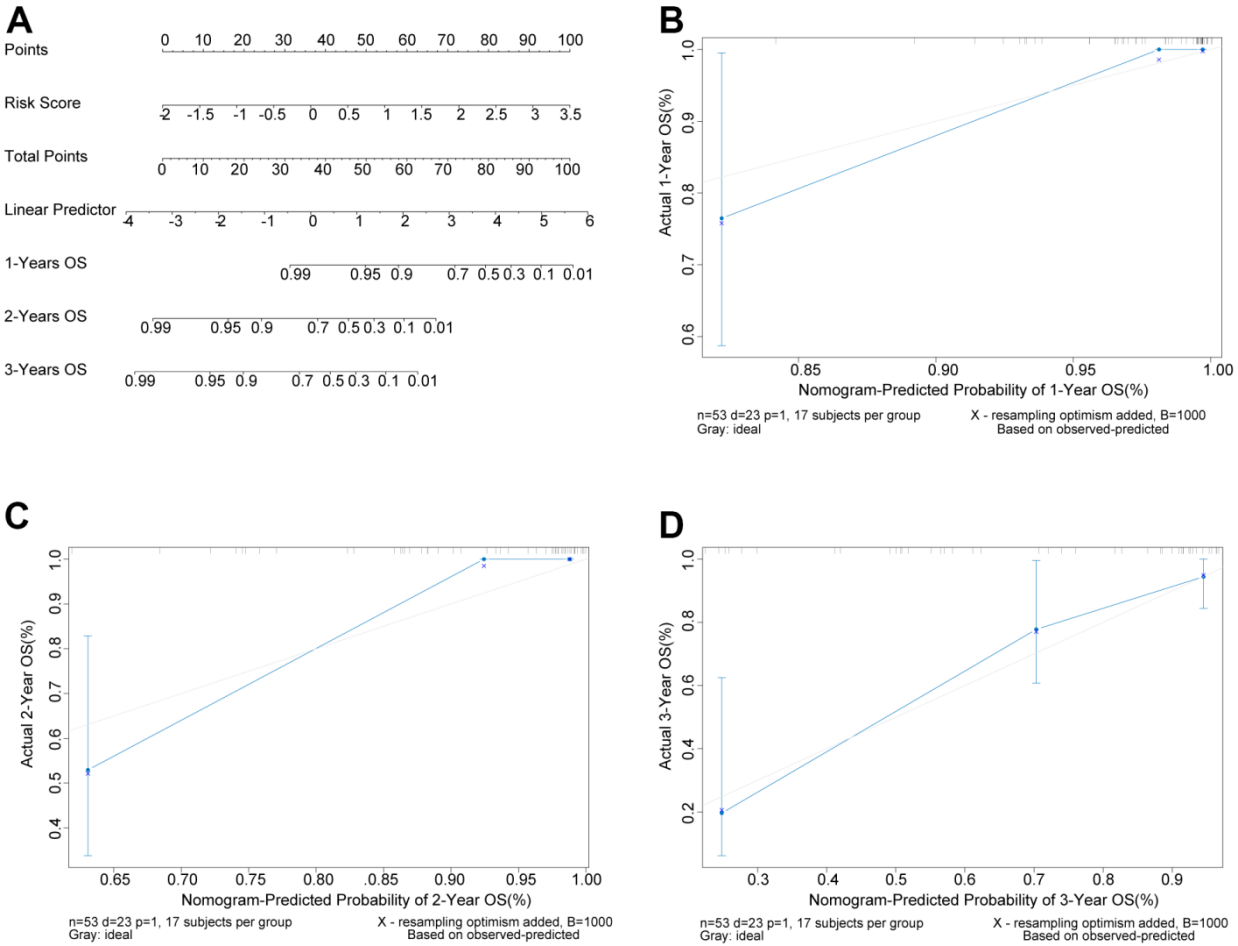
**Nomogram could predict the survival probability of OS patients.** (**A**) Nomogram could predict the 1-year, 2-year, and 3-year overall survival of OS patients. (**B**–**D**) The calibration curves of predicted survival probability of OS patients at 1, 2, and 3 years, separately. X-axis: predicted survival probability; Y-axis: actual survival probability.

Next, we built a Nomogram based on the independent factor Risk score (Figure 4A). For the OS samples, one line was drawn upwards to get the Points from Risk score, which were matched to the Total Points axis accordingly. Then a line drawn downwards the Total Points axis determined the 1-year, 2-year, and 3-year overall survival of OS patients. The calibration curves of predicted survival probability of OS patients relatively well matched the ideal curve (Figure 4B–4D). Our Nomogram displayed a good predictive performance, which also implied the reliability of the Risk score.

### Immune cell infiltration in high and low risk OS patients

**Figure 5:**
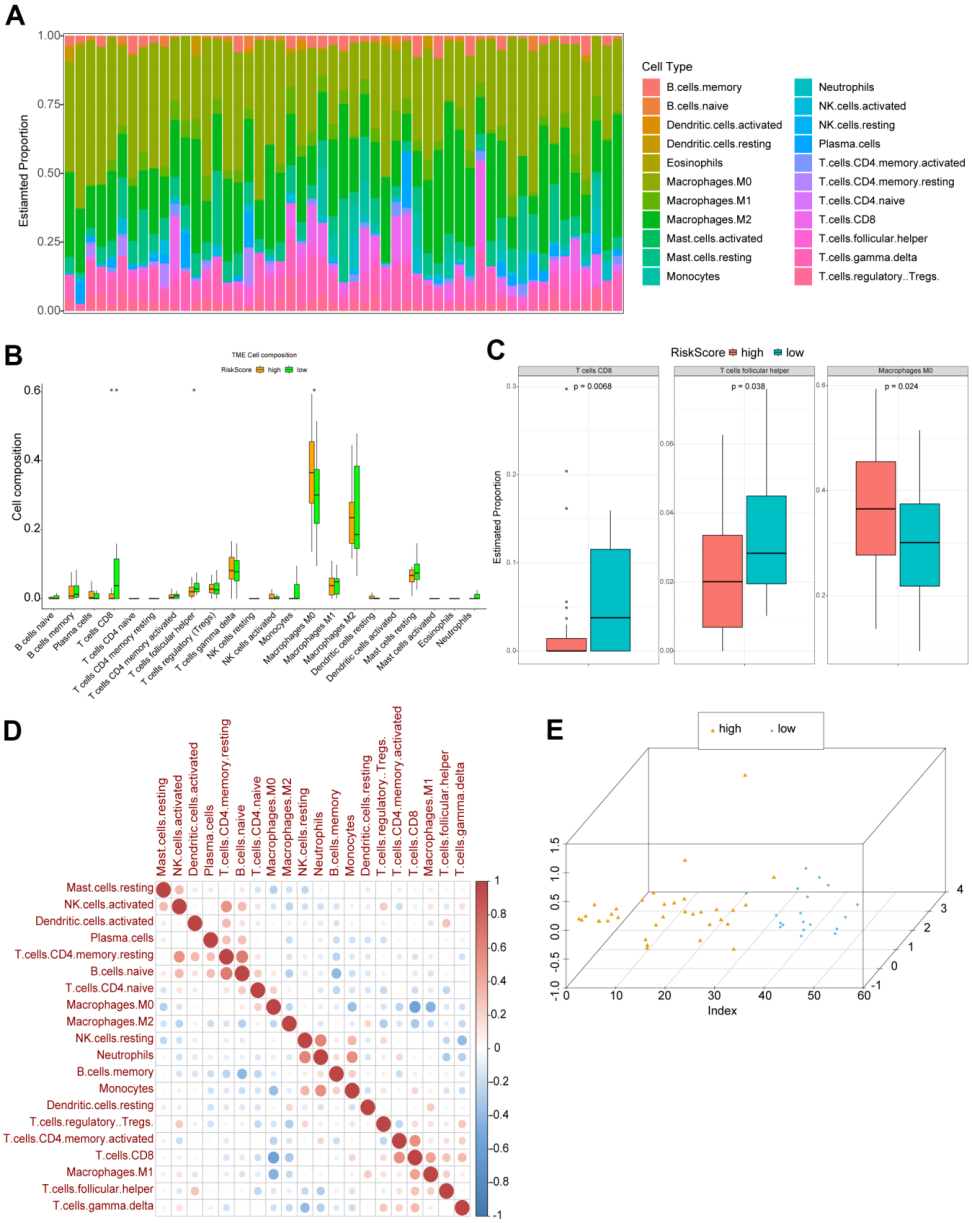
**Immune cell infiltration in high and low risk OS patients.** (**A**) The relative proportions of immune infiltrating cells in OS patients. (**B**) Differentially infiltrated immune cells between high and low risk OS patients. P was calculated with Wilcoxon method. (**C**) Three types of immune cells significantly differentially infiltrated. (**D**) Correlation matrix of various immune cells’ infiltrating proportions. Red: positive correlation; blue: negative correlation. (**E**) The results of PCA.

Basing on CIBERSORT method and LM22 feature matrix, various immune cells’ infiltration was compared between high and low risk OS patients. The immune cell infiltration results of 53 OS patients in GSE21257 dataset (except for 2 abnormal samples) were summarized in Figure 5A, and the differential infiltrating proportions indicated the individual OS patient’s characteristics. Most types of immune cells were differentially infiltrated between high and low risk OS patients (Figure 5B), among which T cells CD8, T cells follicular helper, and Macrophages M0 were significantly differentially infiltrated in high and low risk OS patients (Figure 5C). Whereas, there was a weak correlation among various immune cells’ infiltrating proportions (Figure 5D). The principal component analysis (PCA) based on three significantly differentially infiltrated immune cells suggested that the OS samples could be divided into 2 clusters (Figure 5E).

### Correlation between key immune checkpoints and Risk score

**Figure 6:**
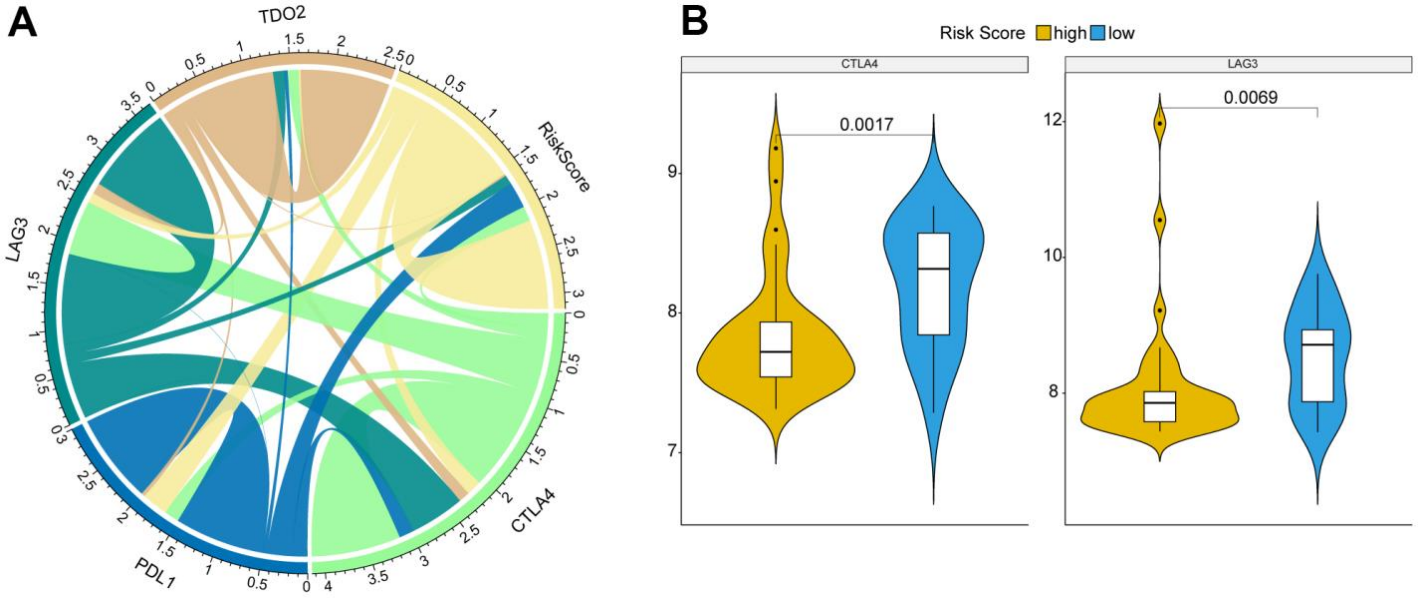
**Correlation between key immune checkpoints and Risk score.** (**A**) Risk score was correlated with immune checkpoints, CTLA4, PDL1, LAG3, and TDO2. (**B**) CTLA4 and LAG3 were significantly differentially expressed between high and low risk OS patients. P was calculated with Wilcoxon method.

Moreover, we analyzed the correlation between several crucial immune checkpoints and Risk score, including CTLA4, PDL1, LAG3, and TDO2. We found that Risk score was correlated with all these immune checkpoints (Figure 6A). CTLA4 and LAG3 were significantly differentially expressed between high and low risk OS patients (Figure 6B).

## DISCUSSION

During the past decades, distant metastasis has limited the prognosis of OS patients [25]. Meanwhile, many studies have evidenced that the apoptosis promotion would inhibit the progression and attenuates the metastasis of OS [26, 27]. Consequently, we herein investigated the potential role of apoptosis-related genes in OS patients, via analyzing the mRNA data and clinical survival information in GEO database. We have firstly constructed and verified a six-gene based prognostic signature for OS patients.

Many factors have been revealed to regulate the cell apoptosis in OS, including miRNAs, proteins, compounds, and so on [28–31]. Considering the vital influence of apoptosis on various tumors, including OS, we firstly downloaded 680 apoptosis-related genes from GEO database. Based on these genes, the OS patients could be divided into 2 clusters with significantly different overall survival, implying the potential effects of apoptosis-related genes on the prognosis of OS. Furthermore, using GSE21257, univariate Cox regression analysis determined 7 prognosis apoptosis-related genes, of which 6 optimal genes were identified. Thus, we have built the Risk score basing on TERT, TRAP1, DNM1L, BAG5, PLEKHF1 and PPP3CB. Our data indicated that in both training set (GSE21257) and validation set (meta-GEO dataset), high risk OS patients had significantly worse overall survival compared with the low risk patients. Not only that, subsequent multivariate Cox regression analysis and Kaplan-Meier survival analysis suggested that high Risk score was an independent poor prognostic factor for OS with various ages or genders.

Meanwhile, some clues have been found to support our prognostic signature based on these 6 genes. TERT (telomerase reverse transcriptase) encodes a catalytic subunit of telomerase, which is influenced by multiple genetic and epigenetic regulations in many cancers [32]. Moreover, TERT has been evidenced to suppress the cisplatin-induced apoptosis in OS cells after cisplatin treatment, which might be a target for overcoming drug resistance [33]. TERT promoter mutations are associated with poorer overall survival of thyroid cancer [34], while the prognostic role of TERT has been seldom reported in OS. Additionally, TRAP1 (TNF receptor associated protein 1) has been indicated to regulated the apoptosis in lung cancer [35] and thyroid carcinoma cells [36], while TRAP1’s role in the prognosis of OS is firstly revealed in our study as far as we know. Whether TRAP1 could regulate the apoptosis and thereby affect the prognosis in OS in a similar way is still unknown, which deserves further exploration. DNM1L has been recently reported as an important prognostic factor for gastric adenocarcinoma [37], involving invasion and apoptosis, which is partly in line with our findings in OS. Despite few studies of PPP3CB in OS, PPP3CB has been evidenced to correlate with the poor prognosis of neuroblastoma [38], while PPP3CB might have conducive effects on pancreatic cancer [39]. Our data provided more insights into PPP3CB in OS, deserving more exploration. As for BAG5 and PLEKHF1, they have not been investigated in OS up to now, while their role in some other cancers have been documented. For example, BAG5 might involve in the invasion of papillary thyroid cancer cells [40]. Besides, BAG5 is indicated to be a suppressor in pancreatic cancer [41]. More details of BAG5 and PLEKHF1 in OS need to be clarified. Collectively, most genes in our prognostic Risk score could be supported by previous studies directly or indirectly. Besides, the good predictive effects of Nomogram based on Risk score also indicated that our prognostic signature was relatively reliable.

Additionally, immunosuppressive microenvironment in OS tissues has been demonstrated basing on immune and stromal cell type information [42]. In our study, three types of immune cells were found to be differentially infiltrated between high and low risk OS patients, comprising T cells CD8, T cells follicular helper, and Macrophages M0, which probably contributed to the different prognosis of OS patients. Among which, stimulating CD8+ cells might inhibit the development of OS [43]. Macrophages M0, M2 have been demonstrated as the most principal infiltrating immune cells in OS [44], which was compatible with our findings. In short, the complex interaction between OS and these immune cell remains to be unclear, which should be studied in the future.

## CONCLUSIONS

To summarize, we have firstly revealed the prognostic role of apoptosis-related genes in OS patients. A six genes-based (TERT, TRAP1, DNM1L, BAG5, PLEKHF1 and PPP3CB) prognostic Risk score signature is probably conducive to distinguish different prognosis of OS patients, which is a promising prognosis prediction alternative for the clinical management.

## Supplementary Material

Supplementary Table 1

## References

[1] Synoradzki KJ, Bartnik E, Czarnecka AM, Fiedorowicz M, Firlej W, Brodziak A, Stasinska A, Rutkowski P, Grieb P. TP53 in Biology and Treatment of Osteosarcoma. Cancers (Basel). 2021; 13:4284. 10.3390/cancers13174284 34503094 PMC8428337

[2] Yang C, Tian Y, Zhao F, Chen Z, Su P, Li Y, Qian A. Bone Microenvironment and Osteosarcoma Metastasis. Int J Mol Sci. 2020; 21:6985. 10.3390/ijms21196985 32977425 PMC7582690

[3] Cortini M, Avnet S, Baldini N. Mesenchymal stroma: Role in osteosarcoma progression. Cancer Lett. 2017; 405:90–9. 10.1016/j.canlet.2017.07.024 28774797

[4] Li YJ, Yao K, Lu MX, Zhang WB, Xiao C, Tu CQ. Prognostic value of the C-reactive protein to albumin ratio: a novel inflammation-based prognostic indicator in osteosarcoma. Onco Targets Ther. 2017; 10:5255–61. 10.2147/OTT.S140560 29138578 PMC5679688

[5] Kayton ML, Huvos AG, Casher J, Abramson SJ, Rosen NS, Wexler LH, Meyers P, LaQuaglia MP. Computed tomographic scan of the chest underestimates the number of metastatic lesions in osteosarcoma. J Pediatr Surg. 2006; 41:200–6. 10.1016/j.jpedsurg.2005.10.024 16410133

[6] Kansara M, Teng MW, Smyth MJ, Thomas DM. Translational biology of osteosarcoma. Nat Rev Cancer. 2014; 14:722–35. 10.1038/nrc3838 25319867

[7] Wang S, Ren T, Huang Y, Bao X, Sun K, Shen D, Guo W. BMPR2 and HIF1-α overexpression in resected osteosarcoma correlates with distant metastasis and patient survival. Chin J Cancer Res. 2017; 29:447–54. 10.21147/j.issn.1000-9604.2017.05.09 29142464 PMC5677136

[8] Mirabello L, Troisi RJ, Savage SA. Osteosarcoma incidence and survival rates from 1973 to 2004: data from the Surveillance, Epidemiology, and End Results Program. Cancer. 2009; 115:1531–43. 10.1002/cncr.24121 19197972 PMC2813207

[9] Misaghi A, Goldin A, Awad M, Kulidjian AA. Osteosarcoma: a comprehensive review. SICOT J. 2018; 4:12. 10.1051/sicotj/2017028 29629690 PMC5890448

[10] Wu CC, Beird HC, Andrew Livingston J, Advani S, Mitra A, Cao S, Reuben A, Ingram D, Wang WL, Ju Z, Hong Leung C, Lin H, Zheng Y, et al. Immuno-genomic landscape of osteosarcoma. Nat Commun. 2020; 11:1008. 10.1038/s41467-020-14646-w 32081846 PMC7035358

[11] Rosemann M, Gonzalez-Vasconcellos I, Domke T, Kuosaite V, Schneider R, Kremer M, Favor J, Nathrath M, Atkinson MJ. A Rb1 promoter variant with reduced activity contributes to osteosarcoma susceptibility in irradiated mice. Mol Cancer. 2014; 13:182. 10.1186/1476-4598-13-182 25092376 PMC4237942

[12] Wu ZL, Deng YJ, Zhang GZ, Ren EH, Yuan WH, Xie QQ. Development of a novel immune-related genes prognostic signature for osteosarcoma. Sci Rep. 2020; 10:18402. 10.1038/s41598-020-75573-w 33110201 PMC7591524

[13] Xiao B, Liu L, Li A, Xiang C, Wang P, Li H, Xiao T. Identification and Verification of Immune-Related Gene Prognostic Signature Based on ssGSEA for Osteosarcoma. Front Oncol. 2020; 10:607622. 10.3389/fonc.2020.607622 33384961 PMC7771722

[14] Shi Y, He R, Zhuang Z, Ren J, Wang Z, Liu Y, Wu J, Jiang S, Wang K. A risk signature-based on metastasis-associated genes to predict survival of patients with osteosarcoma. J Cell Biochem. 2020; 121:3479–90. 10.1002/jcb.29622 31898371

[15] McIlwain DR, Berger T, Mak TW. Caspase functions in cell death and disease. Cold Spring Harb Perspect Biol. 2013; 5:a008656. 10.1101/cshperspect.a008656 23545416 PMC3683896

[16] Strasser A, O’Connor L, Dixit VM. Apoptosis signaling. Annu Rev Biochem. 2000; 69:217–45. 10.1146/annurev.biochem.69.1.217 10966458

[17] Elmore S. Apoptosis: a review of programmed cell death. Toxicol Pathol. 2007; 35:495–516. 10.1080/01926230701320337 17562483 PMC2117903

[18] Carneiro BA, El-Deiry WS. Targeting apoptosis in cancer therapy. Nat Rev Clin Oncol. 2020; 17:395–417. 10.1038/s41571-020-0341-y 32203277 PMC8211386

[19] Liu K, Ren T, Huang Y, Sun K, Bao X, Wang S, Zheng B, Guo W. Apatinib promotes autophagy and apoptosis through VEGFR2/STAT3/BCL-2 signaling in osteosarcoma. Cell Death Dis. 2017; 8:e3015. 10.1038/cddis.2017.422 28837148 PMC5596600

[20] Li B, Zhou P, Xu K, Chen T, Jiao J, Wei H, Yang X, Xu W, Wan W, Xiao J. Metformin induces cell cycle arrest, apoptosis and autophagy through ROS/JNK signaling pathway in human osteosarcoma. Int J Biol Sci. 2020; 16:74–84. 10.7150/ijbs.33787 31892847 PMC6930379

[21] Yang ZM, Yang MF, Yu W, Tao HM. Molecular mechanisms of estrogen receptor β-induced apoptosis and autophagy in tumors: implication for treating osteosarcoma. J Int Med Res. 2019; 47:4644–55. 10.1177/0300060519871373 31526167 PMC6833400

[22] Chen W, Liu Q, Fu B, Liu K, Jiang W. Overexpression of GRIM-19 accelerates radiation-induced osteosarcoma cells apoptosis by p53 stabilization. Life Sci. 2018; 208:232–8. 10.1016/j.lfs.2018.07.015 30005830

[23] Friedman J, Hastie T, Tibshirani R. Regularization Paths for Generalized Linear Models via Coordinate Descent. J Stat Softw. 2010; 33:1–22. 20808728 PMC2929880

[24] Newman AM, Liu CL, Green MR, Gentles AJ, Feng W, Xu Y, Hoang CD, Diehn M, Alizadeh AA. Robust enumeration of cell subsets from tissue expression profiles. Nat Methods. 2015; 12:453–7. 10.1038/nmeth.3337 25822800 PMC4739640

[25] Lv YF, Yan GN, Meng G, Zhang X, Guo QN. Enhancer of zeste homolog 2 silencing inhibits tumor growth and lung metastasis in osteosarcoma. Sci Rep. 2015; 5:12999. 10.1038/srep12999 26265454 PMC4533017

[26] Li R, Shi Y, Zhao S, Shi T, Zhang G. NF-κB signaling and integrin-β1 inhibition attenuates osteosarcoma metastasis via increased cell apoptosis. Int J Biol Macromol. 2019; 123:1035–43. 10.1016/j.ijbiomac.2018.11.003 30399378

[27] Zhang Y, Weng Q, Chen J, Han J. Morusin Inhibits Human Osteosarcoma via the PI3K-AKT Signaling Pathway. Curr Pharm Biotechnol. 2020; 21:1402–9. 10.2174/1389201021666200416093457 32297574

[28] Li J, Yang Z, Li Y, Xia J, Li D, Li H, Ren M, Liao Y, Yu S, Chen Y, Yang Y, Zhang Y. Cell apoptosis, autophagy and necroptosis in osteosarcoma treatment. Oncotarget. 2016; 7:44763–78. 10.18632/oncotarget.8206 27007056 PMC5190133

[29] Song B, Wang Y, Xi Y, Kudo K, Bruheim S, Botchkina GI, Gavin E, Wan Y, Formentini A, Kornmann M, Fodstad O, Ju J. Mechanism of chemoresistance mediated by miR-140 in human osteosarcoma and colon cancer cells. Oncogene. 2009; 28:4065–74. 10.1038/onc.2009.274 19734943 PMC2783211

[30] Maugg D, Rothenaigner I, Schorpp K, Potukuchi HK, Korsching E, Baumhoer D, Hadian K, Smida J, Nathrath M. New small molecules targeting apoptosis and cell viability in osteosarcoma. PLoS One. 2015; 10:e0129058. 10.1371/journal.pone.0129058 26039064 PMC4454490

[31] Li M, Zhu Y, Zhang H, Li L, He P, Xia H, Zhang Y, Mao C. Delivery of inhibitor of growth 4 (ING4) gene significantly inhibits proliferation and invasion and promotes apoptosis of human osteosarcoma cells. Sci Rep. 2014; 4:7380. 10.1038/srep07380 25490312 PMC4260466

[32] Dratwa M, Wysoczańska B, Łacina P, Kubik T, Bogunia-Kubik K. TERT-Regulation and Roles in Cancer Formation. Front Immunol. 2020; 11:589929. 10.3389/fimmu.2020.589929 33329574 PMC7717964

[33] Zhang Z, Yu L, Dai G, Xia K, Liu G, Song Q, Tao C, Gao T, Guo W. Telomerase reverse transcriptase promotes chemoresistance by suppressing cisplatin-dependent apoptosis in osteosarcoma cells. Sci Rep. 2017; 7:7070. 10.1038/s41598-017-07204-w 28765565 PMC5539325

[34] Chung JH. BRAF and TERT promoter mutations: clinical application in thyroid cancer. Endocr J. 2020; 67:577–84. 10.1507/endocrj.EJ20-0063 32321884

[35] Zhang X, Dong Y, Gao M, Hao M, Ren H, Guo L, Guo H. Knockdown of TRAP1 promotes cisplatin-induced apoptosis by promoting the ROS-dependent mitochondrial dysfunction in lung cancer cells. Mol Cell Biochem. 2021; 476:1075–82. 10.1007/s11010-020-03973-7 33196942

[36] Palladino G, Notarangelo T, Pannone G, Piscazzi A, Lamacchia O, Sisinni L, Spagnoletti G, Toti P, Santoro A, Storto G, Bufo P, Cignarelli M, Esposito F, Landriscina M. TRAP1 regulates cell cycle and apoptosis in thyroid carcinoma cells. Endocr Relat Cancer. 2016; 23:699–709. 10.1530/ERC-16-0063 27422900

[37] Xu XW, Yang XM, Zhao WJ, Zhou L, Li DC, Zheng YH. DNM1L, a key prognostic predictor for gastric adenocarcinoma, is involved in cell proliferation, invasion, and apoptosis. Oncol Lett. 2018; 16:3635–41. 10.3892/ol.2018.9138 30127972 PMC6096219

[38] Shakhova I, Li Y, Yu F, Kaneko Y, Nakamura Y, Ohira M, Izumi H, Mae T, Varfolomeeva SR, Rumyantsev AG, Nakagawara A. PPP3CB contributes to poor prognosis through activating nuclear factor of activated T-cells signaling in neuroblastoma. Mol Carcinog. 2019; 58:426–35. 10.1002/mc.22939 30457174

[39] Hang J, Lau SY, Yin R, Zhu L, Zhou S, Yuan X, Wu L. The role of phosphoprotein phosphatases catalytic subunit genes in pancreatic cancer. Biosci Rep. 2021; 41:BSR20203282. 10.1042/BSR20203282 33270085 PMC7785039

[40] Zhang DL, Wang JM, Wu T, Du X, Yan J, Du ZX, Wang HQ. BAG5 promotes invasion of papillary thyroid cancer cells via upregulation of fibronectin 1 at the translational level. Biochim Biophys Acta Mol Cell Res. 2020; 1867:118715. 10.1016/j.bbamcr.2020.118715 32275930

[41] Yu Y, Liu L, Ma R, Gong H, Xu P, Wang C. MicroRNA-127 is aberrantly downregulated and acted as a functional tumor suppressor in human pancreatic cancer. Tumour Biol. 2016; 37:14249–57. 10.1007/s13277-016-5270-0 27571739

[42] Zhou Y, Yang D, Yang Q, Lv X, Huang W, Zhou Z, Wang Y, Zhang Z, Yuan T, Ding X, Tang L, Zhang J, Yin J, et al. Single-cell RNA landscape of intratumoral heterogeneity and immunosuppressive microenvironment in advanced osteosarcoma. Nat Commun. 2020; 11:6322. 10.1038/s41467-020-20059-6 33303760 PMC7730477

[43] Yahiro K, Matsumoto Y, Yamada H, Endo M, Setsu N, Fujiwara T, Nakagawa M, Kimura A, Shimada E, Okada S, Oda Y, Nakashima Y. Activation of TLR4 signaling inhibits progression of osteosarcoma by stimulating CD8-positive cytotoxic lymphocytes. Cancer Immunol Immunother. 2020; 69:745–58. 10.1007/s00262-020-02508-9 32047957 PMC11027819

[44] Niu J, Yan T, Guo W, Wang W, Zhao Z, Ren T, Huang Y, Zhang H, Yu Y, Liang X. Identification of Potential Therapeutic Targets and Immune Cell Infiltration Characteristics in Osteosarcoma Using Bioinformatics Strategy. Front Oncol. 2020; 10:1628. 10.3389/fonc.2020.01628 32974202 PMC7471873

