# Supplementary material for: Apoptosis-related genes-based prognostic signature for osteosarcoma
Source: Aging (Albany NY). 2022 May 3;14(9):3813–25. doi: 10.18632/aging.204042 (PMC9134960; doi:10.18632/aging.204042)
Supplement: Supplementary Table 1 [file aging-14-204042-s001.docx]

**Supplementary Table 1. Apoptosis_GeneID.**

| AIFM1 | ATF4 | FASTK | MOAP1 | SGPP1 |
| --- | --- | --- | --- | --- |
| AKT1 | ATP2A1 | FBH1 | MPV17L | SH3RF1 |
| AKT2 | ATP2A3 | FBXW7 | MSH2 | SHH |
| AKT3 | ATP5IF1 | FEM1B | MSH6 | SHISA5 |
| APAF1 | AVP | FGA | MSX1 | SIAH1 |
| ATM | BAG3 | FGB | MUC1 | SIAH2 |
| BAD | BAG5 | FGF10 | MUL1 | SIRT1 |
| BAX | BAG6 | FGFR1 | NACC2 | SIVA1 |
| BCL2 | BAK1 | FGFR3 | NANOS3 | SKIL |
| BCL2L1 | BBC3 | FGG | NBN | SLC25A5 |
| BID | BCAP31 | FHIT | NCK1 | SLC35F6 |
| BIRC2 | BCL10 | FIGNL1 | NCK2 | SLC9A3R1 |
| BIRC3 | BCL2A1 | FIS1 | NDUFA13 | SMAD3 |
| CAPN1 | BCL2L10 | FNIP2 | NDUFS3 | SNAI1 |
| CAPN2 | BCL2L11 | FXN | NFATC4 | SNW1 |
| CASP10 | BCL2L12 | FYN | NFE2L2 | SOD1 |
| CASP3 | BCL2L14 | FZD9 | NGFR | SOD2 |
| CASP6 | BCL2L2 | G0S2 | NKX3-1 | SORT1 |
| CASP7 | BCL3 | GABARAP | NLE1 | SP100 |
| CASP8 | BCLAF1 | GATA1 | NME5 | SRPX |
| CASP9 | BDKRB2 | GATA4 | NMT1 | SST |
| CFLAR | BDNF | GCLM | NOC2L | SSTR3 |
| CHP1 | BECN1 | GDNF | NOG | ST20 |
| CHP2 | BIK | GFRAL | NOL3 | STK24 |
| CHUK | BIRC6 | GGCT | NONO | STK25 |
| CSF2RB | BLOC1S2 | GHITM | NOS3 | STK3 |
| CYCS | BMP4 | GNAI2 | NOX1 | STK4 |
| DFFA | BMP5 | GNAI3 | NR4A2 | STRADB |
| DFFB | BMPR1B | GPER1 | NUPR1 | STX4 |
| ENDOD1 | BNIP3 | GPX1 | OPA1 | STYXL1 |
| ENDOG | BNIP3L | GRINA | P2RX4 | SYVN1 |
| EXOG | BOK | GSDME | P2RX7 | TAF9 |
| FADD | BRCA1 | GSK3A | P4HB | TAF9B |
| FAS | BRCA2 | GSK3B | PAK2 | TCF7L2 |
| FASLG | BRSK2 | GSKIP | PAK5 | TERT |
| IKBKB | BTK | GSTP1 | PARK7 | TFDP2 |
| IKBKG | CAAP1 | GZMB | PARP1 | TFPT |
| IL1A | CASP1 | HDAC1 | PARP2 | TGFB1 |
| IL1B | CASP12 | HERPUD1 | PAWR | TGFB2 |
| IL1R1 | CASP2 | HGF | PCGF2 | TGFBR1 |
| IL1RAP | CASP4 | HIC1 | PDCD10 | THBS1 |
| IL3 | CASP5 | HIF1A | PDCD5 | TICAM1 |
| IL3RA | CASP8AP2 | HINT1 | PDCD6 | TICAM2 |
| IRAK1 | CCAR2 | HIP1 | PDIA3 | TIMM50 |
| IRAK2 | CCK | HIP1R | PDK1 | TIMP3 |
| IRAK3 | CD14 | HIPK1 | PDK2 | TLR3 |
| IRAK4 | CD24 | HIPK2 | PDPK1 | TLR4 |
| MAP3K14 | CD27 | HMGB2 | PDX1 | TM2D1 |
| MYD88 | CD28 | HMOX1 | PEA15 | TMBIM1 |
| NFKB1 | CD38 | HNRNPK | PELI3 | TMBIM6 |
| NFKBIA | CD3E | HRAS | PERP | TMC8 |
| NGF | CD44 | HRK | PF4 | TMEM102 |
| NTRK1 | CD5 | HSPA1A | PHIP | TMEM109 |
| PIK3CA | CD70 | HSPA1B | PHLDA3 | TMEM117 |
| PIK3CB | CD74 | HSPB1 | PIAS4 | TMEM14A |
| PIK3CD | CDIP1 | HTRA2 | PIDD1 | TMEM161A |
| PIK3CG | CDKN1A | HTT | PIH1D1 | TNFAIP3 |
| PIK3R1 | CDKN2D | HYAL2 | PINK1 | TNFRSF12A |
| PIK3R2 | CEBPB | HYOU1 | PLAGL2 | TNFRSF1B |
| PIK3R3 | CHAC1 | ICAM1 | PLAUR | TNFRSF25 |
| PIK3R5 | CHCHD10 | IFI16 | PLEKHF1 | TNFSF12 |
| PPP3CA | CIB1 | IFI27 | PLSCR3 | TOPORS |
| PPP3CB | CIDEB | IFI27L1 | PMAIP1 | TP53BP2 |
| PPP3CC | CLU | IFI27L2 | PML | TP63 |
| PPP3R1 | COA8 | IFI6 | POLB | TP73 |
| PPP3R2 | COL2A1 | IFNB1 | POU4F1 | TPD52L1 |
| PRKACA | CRADD | IFNG | POU4F2 | TPT1 |
| PRKACB | CREB3 | IGF1 | PPARD | TRAF1 |
| PRKACG | CREB3L1 | IKBKE | PPIA | TRAF7 |
| PRKAR1A | CRH | IL12A | PPIF | TRAP1 |
| PRKAR1B | CRIP1 | IL19 | PPM1F | TRIAP1 |
| PRKAR2A | CSF2 | IL2 | PPP1CA | TRIB3 |
| PRKAR2B | CSNK2A1 | IL20RA | PPP1R13B | TRIM32 |
| PRKX | CSNK2A2 | IL33 | PPP1R15A | TRIM39 |
| RELA | CTH | IL4 | PPP2R1B | TXNDC12 |
| RIPK1 | CTNNA1 | IL6R | PRDX2 | TYROBP |
| TNF | CTSC | IL7 | PRELID1 | UACA |
| TNFRSF10A | CTTN | INCA1 | PRKCA | UBB |
| TNFRSF10B | CUL1 | ING2 | PRKCD | UBE2K |
| TNFRSF10C | CUL2 | ING5 | PRKDC | UBE4B |
| TNFRSF10D | CUL3 | INHBA | PRKN | UBQLN1 |
| TNFRSF1A | CUL4A | INHBB | PRKRA | UMOD |
| TNFSF10 | CUL5 | INS | PRODH | UNC5B |
| TP53 | CX3CL1 | ITGA6 | PSEN1 | URI1 |
| TRADD | CX3CR1 | ITGAM | PSMD10 | USP28 |
| TRAF2 | CXCL12 | ITGAV | PSME3 | USP47 |
| XIAP | CYLD | ITM2C | PTEN | VDAC2 |
| ANKRD13C | CYP1B1 | ITPR1 | PTGIS | VNN1 |
| BMF | DAB2IP | ITPRIP | PTH | WDR35 |
| BRMS1 | DAP | IVNS1ABP | PTPMT1 | WNT4 |
| CAV1 | DAP3 | JAK2 | PTPN1 | WWOX |
| CEACAM5 | DAPK1 | JMY | PTPN2 | XBP1 |
| CEACAM6 | DAPK3 | JUN | PTPRC | YAP1 |
| CHEK2 | DAPL1 | KDM1A | PTTG1IP | YBX3 |
| CRYBA1 | DAXX | KITLG | PYCARD | YWHAB |
| DAPK2 | DBH | KRT18 | QARS1 | YWHAE |
| E2F1 | DCC | KRT8 | RACK1 | YWHAG |
| ITGA5 | DDIAS | LCK | RAF1 | YWHAH |
| ITGB1 | DDIT3 | LGALS12 | RB1 | YWHAQ |
| MAP3K7 | DDIT4 | LGALS3 | RB1CC1 | YWHAZ |
| MCL1 | DDX3X | LRRK2 | RBCK1 | ZC3HC1 |
| MTOR | DDX47 | LTBR | RET | ZDHHC3 |
| MYBBP1A | DDX5 | LY96 | RFFL | ZMYND11 |
| NOTCH1 | DEDD | MADD | RHOT1 | ZNF205 |
| NTRK2 | DEDD2 | MAEL | RHOT2 | ZNF385A |
| PDK4 | DELE1 | MAGEA3 | RIPK3 | ZNF385B |
| PTK2 | DEPTOR | MAP2K5 | RNF183 | ZNF622 |
| PTRH2 | DIABLO | MAP3K5 | RNF186 | ZSWIM2 |
| SIK1 | DIDO1 | MAPK7 | RNF34 | ANLN |
| SNAI2 | DNAJA1 | MAPK8 | RNF41 | ANO6 |
| SRC | DNAJC10 | MAPK8IP1 | RPL11 | EMP1 |
| STK11 | DNM1L | MAPK8IP2 | RPL26 | EMP2 |
| TFDP1 | DPF2 | MAPK9 | RPS27L | EMP3 |
| TLE1 | DYRK2 | MARCHF7 | RPS3 | LPAR1 |
| TLE5 | E2F2 | MAZ | RPS6KB1 | LPAR3 |
| TSC2 | EDA2R | MDM2 | RPS7 | MYLK |
| ZNF304 | EIF2AK3 | MELK | RRP8 | PMP22 |
| AATF | ELL3 | MFF | RTKN2 | DTX3L |
| ABL1 | ENO1 | MIF | RTL10 | FAM220A |
| ACAA2 | EP300 | MIR132 | S100A8 | FAM220BP |
| ACKR3 | EPHA2 | MIR15A | S100A9 | H2BC9 |
| ACVR1 | EPO | MIR16-1 | SCG2 | HSF1 |
| ACVR1B | ERBB3 | MIR17 | SCN2A | PARP9 |
| ADORA1 | ERCC6 | MIR198 | SCRT2 | PTPRT |
| AEN | ERN1 | MIR21 | SELENOK | FSTL4 |
| AGT | ERN2 | MIR210 | SELENOS | FURIN |
| AGTR2 | ERO1A | MIR221 | SENP1 | HAP1 |
| ANXA6 | ERP29 | MIR222 | SEPTIN4 | NTRK3 |
| APPL1 | EYA1 | MIR26B | SERINC3 | PCSK6 |
| AR | EYA2 | MIR27B | SERPINE1 | IRGM |
| ARHGEF2 | EYA3 | MIR449A | SFN | FHOD1 |
| ARL6IP5 | EYA4 | MKNK2 | SFPQ | FMNL1 |
| ARMC10 | FAF1 | MLH1 | SFRP1 | PANX1 |
| ARRB2 | FAIM | MLLT11 | SFRP2 | ROCK1 |
| ASAH2 | FAIM2 | MMP9 | SGMS1 | SDF4 |
| ATF3 | FAM162A | MNT | SGPL1 | TPM1 |
